# “Light” on Phototherapy—Complications and Strategies for Shortening Its Duration, A Review of the Literature

**DOI:** 10.3390/children10101699

**Published:** 2023-10-17

**Authors:** Irit Shoris, Ayala Gover, Arina Toropine, Adir Iofe, Rasha Zoabi-Safadi, Svetlana Tsuprun, Arieh Riskin

**Affiliations:** 1 Department of Neonatology, Bnai Zion Medical Center, 47 Golomb Street, P.O. Box 4940, Haifa 31048, Israel; 2 Rappaport Faculty of Medicine, Technion—Israel Institute of Technology, P.O. Box 9697, Haifa 32000, Israel; irit.shoris@b-zion.org.il (I.S.); ayala.gover@b-zion.org.il (A.G.); arina.toropine@b-zion.org.il (A.T.); adir.iofe@b-zion.org.il (A.I.); rasha.safadi@b-zion.org.il (R.Z.-S.); svetlana.tsuprun@b-zion.org.il (S.T.)

**Keywords:** neonatal hyperbilirubinemia (NHB), phototherapy, bilirubin

## Abstract

Neonatal hyperbilirubinemia is an extremely common metabolic complication of the neonatal period which may be associated with bilirubin encephalopathy and even death. Adverse neurological consequences are preventable if a timely diagnosis and treatment are provided. Phototherapy is usually the preferred option to decrease hyperbilirubinemia. Although considered to be safe, evidence in recent years has shown that this treatment may not be free of side effects and short- and long-term unfavorable outcomes. These are usually mild or rare, but should be decreased or avoided if possible. Many useful complementary measures and treatments have been described that could shorten the duration of exposure to phototherapy. However, there is no current unequivocal recommendation to use any of the methods presented in this review. Our review aims to depict the wide range of possible complementary treatments to phototherapy, and to provide the scientific and clinical evidence available regarding their usefulness. It is essential that, while utilizing the full potential of phototherapy to treat hyperbilirubinemia, caregivers are aware of its side effects and possible inherent dangers, and seek ways to minimize the exposure to phototherapy to what is really needed for the newborn. Further studies are needed to clarify the preferred complementary treatments that could reduce the duration of exposure to phototherapy without impairing its effectiveness.

## 1. Introduction

Neonatal hyperbilirubinemia (NHB) is an extremely common metabolic complication in newborns, affecting over 60% of term infants and around 80% of preterm infants in the first days of life. Approximately 10% of breastfed infants have persistent jaundice during the first few weeks after birth [1,2,3,4]. Neonatal jaundice presents with yellowish discoloration evident on the skin, and in mucous membranes and sclera. Initiating the treatment of neonatal jaundice, whether physiological or pathological, is usually guided by elevated serum unconjugated bilirubin levels beyond the normal range [5]. Untreated hyperbilirubinemia is a major health problem. The guidelines for the management of hyperbilirubinemia in newborn babies at 35 weeks of gestation or more have recently been updated by the American Academy of Pediatrics [6]. Several factors have been found to be associated with hyperbilirubinemia in the newborn period. The primary causes of hyperbilirubinemia of the newborn include increased bilirubin production due to hemolysis, infection, decreased bilirubin clearance related to intestinal reabsorption or genetic factors, and maternal factors such as maternal diabetes, etc. Other common causes are related to breastfeeding and breastmilk. Interestingly, studies from the last decade have examined the relationship between fetal exposure and air pollution and the relationship with postpartum morbidity. Associations between in utero exposure to high concentrations of specific air pollutants (CO, SO_2_, O_3_, PM10, PM2.5, NO, NO_2_, NMHC, and CH4) and a higher risk of developing hyperbilirubinemia were found [7,8]. Similarly, other studies have shown a relationship between exposure to higher concentrations of specific air pollutants and a greater risk of postnatal phototherapy treatment [9]. In addition, an association between maternal smoking and increased bilirubin levels in the first three days of life was found [10]. The relationship described between endometriosis or placental suffering and increased bilirubin levels [11] could be explained by the same underlying mechanism as that related to increased hemoglobin levels in utero in response to relative hypoxia, followed by excessive breakdown after delivery, leading to increased bilirubin levels. The Global Burden of Disease Study ranked severe neonatal hyperbilirubinemia among the 10 leading causes for neonatal death in countries with poorly developed medical systems and high rates of neonatal mortality [12]. However, these poor outcomes are observed, although less frequently, in industrialized countries as well. Reports from the United States, Canada, and Europe indicate that neonatal jaundice with severe hyperbilirubinemia is considered an unsolved problem even in the current era, despite significant progress in knowledge, treatment, and technology [13].

## 2. Materials and Methods

Our review aims to describe the broad topic of the available possibilities for complementary treatments to phototherapy. After discussing the pathophysiology of NHB and its treatment, focusing on phototherapy and its possible side effects and short- and long-term adverse outcomes, we thoroughly review the current available literature on possible complementary treatments that could decrease exposure to phototherapy without compromising its therapeutic effects.

## 3. Neonatal Jaundice and Phototherapy

### 3.1. Risk Factors for Neonatal Jaundice

Several factors were found to be associated with hyperbilirubinemia in the neonatal period. Various etiologies include: (a) increased bilirubin production: isoimmune hemolytic disease due to the incompatibility of ABO blood groups or Rh isoimmunization, some cases of glucose-6-phosphate-dehydrogenase (G6PD) deficiency, infection, hemolysis, cephalohematoma, and internal bleeding; (b) decreased bilirubin clearance: genetic polymorphisms, such as UDP-glucuronosyltransferase (UGT 1A) and many cases of glucose-6-phosphate-dehydrogenase (G6PD) deficiency and intestinal obstruction; and (c) miscellaneous or multiple causes such as maternal diabetes, congenital hypothyroidism, drugs (e.g., sulfa, ceftriaxone and penicillins), breast milk jaundice, breastfeeding, preterm birth, hypoxia, acidosis, hypoglycemia, and east Asian origin [13,14]. Other factors that may play a role are male sex, cesarean delivery, and a maternal age over 30 [15]. Although NHB, regardless of cause, may require phototherapy if bilirubin levels rise beyond certain levels, in order to prevent bilirubin encephalopathy, identifying the primary cause is of utmost importance in dictating treatment modalities, e.g., if sepsis is the primary cause for the exacerbation of neonatal jaundice, antibiotics are the primary treatment.

### 3.2. Risk Factors for Bilirubin Induced Brain Toxicity

A low level of albumin is one risk factor for bilirubin-induced brain toxicity and encephalopathy. The reduced binding capacity of albumin increases unconjugated bilirubin transfer across the blood–brain barrier. The administration of albumin reduces this risk, and in cases of hypoalbuminemia, the administration of albumin should be considered as an adjunct treatment to phototherapy, regardless of the primary cause of NHB. Recently, a cord blood albumin level of less than 2.8 g/dL was shown to be significantly correlated with hyperbilirubinemia requiring early treatment, while cord blood albumin above 3.3 g/dL was associated with safe early discharge [2]. In another study including 210 term and preterm infants, cord blood bilirubin levels predicted significant serum hyperbilirubinemia at 48 h. These markers could guide the early initiation of phototherapy in newborns at risk, thus reducing the morbidity and mortality associated with severe hyperbilirubinemia [16].

### 3.3. Severe NHB and Bilirubin Encephalopathy

Infants developing complications of severe NHB frequently have total serum bilirubin (TSB) levels above 20 to 25 mg/dL, which are associated with both acute bilirubin encephalopathy (ABE) and chronic bilirubin encephalopathy (CBE), also termed Kernicterus Spectrum Disorder (KSD). These high bilirubin levels may necessitate an exchange transfusion (ET) and may lead to jaundice-related death [17,18]. Excessive free, unconjugated bilirubin (UCB) in the newborn’s bloodstream is able to cross the blood–brain barrier and cause injury to the central nervous system, selectively damaging vulnerable target neurons in the basal ganglia and cerebellum, ultimately leading to cerebral palsy, visual impairment, and hearing deficiencies. Most survivors of acute bilirubin encephalopathy have unfavorable outcomes in childhood, such as cerebral palsy and sensory-neural hearing loss [2,13]. Adverse neurological consequences are preventable if a prompt diagnosis and treatment are given. In high-income countries, long-term complications from hyperbilirubinemia rarely occur. Recent years have shown an encouraging trend regarding the early diagnosis and treatment of NHB in low- and middle-income countries. Education and the use of more advanced technologies have contributed greatly to this improvement. The existing difficulties are probably still related to issues of accessibility to medical services and maintaining continuity of care [18]. It is important, however, to remember that a certain level of bilirubin in the newborn’s blood could be beneficial due to the protective antioxidant effect of bilirubin on cell membranes. Under the influence of phototherapy, bilirubin levels decrease, and this may weaken this protective effect and make cell membranes more sensitive to damage [19].

### 3.4. Common Treatments of NHB

Standard therapeutic interventions, including proper hydration, phototherapy, medications, and exchange transfusion (ET) are used to reduce elevated bilirubin levels. Phototherapy is usually the preferred choice for preventing possible encephalopathy. This treatment for newborn babies was first tried in the 1950s by the nurse Jean Ward at Rochford General Hospital in Essex, England. She realized that sunlight reduces jaundice in newborns. This discovery led to the development of phototherapy, which is probably the most common treatment applied to newborn babies. The first work published on the subject tried to explain the relationship between high bilirubin and kernicterus and the effect of light on lowering bilirubin levels [20]. Since then, it has altered our approach to treating infants with hyperbilirubinemia and has saved millions of lives from death and disability. In the last decade, progress has been made in understanding how phototherapy works, its practical applications, and its complications [5,21].

The excessive photon exposure during phototherapy has raised many concerns regarding the oxidative stress absorbed through the skin of infants, especially low-birth-weight premature infants treated with intensive phototherapy. Many studies on the possible adverse effects of phototherapy on newborns have been published over the past few years. In light of the new understanding of its possible side effects, especially long-term effects, phototherapy has been recently regarded as a “medication with a strict and careful dosing” and research has been conducted to find new therapeutic alternatives [5,22].

### 3.5. Mechanisms of Phototherapy

Bilirubin best absorbs light at a 458 nm wavelength, so the best phototherapy is provided with blue light (450–470 nm) [22]. Irradiation generates a photochemical reaction in the extravascular space of the skin, transforming bilirubin into water-soluble isomers that bypass the liver and can be more easily eliminated in the bile, thus reducing bilirubin levels. A higher illuminated area increases its effectiveness. This process, known as photo isomerization, involves a light-induced transformation of the natural isomer 4Z, 15Z into the polar hydrosoluble 4Z, 15E isomer. Fast re-isomerization, especially after the cessation of phototherapy, may lead to the reabsorption of unconjugated bilirubin and a “rebound” phenomenon. Phototherapy also causes structural isomerization, although to a much lesser extent. This involves cyclization to lumirubin, which is irreversible, is excreted in the bile and urine, and cannot be reabsorbed [23].

### 3.6. Types of Phototherapy Devices

The first artificial light device in use was prepared at Rochford General Hospital. It consisted of eight fluorescent tubes suspended above a cradle with the option to turn on the lights individually to vary the amount of power supplied [20]. Today, there are different types of devices for delivering phototherapy, including fluorescent lights (wavelength range of 400–520 nm), quartz halogen lamps, fiber optic blankets, or light-emitting diodes (LEDs) [24]. In general, the purpose is to administer intensive phototherapy to the largest body surface area possible. Fluorescent tubes and halogen lamps were used in the past as the light source for phototherapy. According to the new AAP 2022 guidelines, during intensive phototherapy, narrow-spectrum blue LED light with radiation of at least 30 µW/cm^2^ per nanometer at a wavelength around 475 nm should be used. Light other than that between the range of 460 and 490 nm causes unnecessary heat and possibly harmful wavelengths [25]. A light-emitting diode (LED wavelength is 460–490 nm) is a newer type of light source which is energy-efficient, has a longer life, has a low heat output, is effective in lowering bilirubin levels, and is thus recommended for better clinical effectiveness, with a shorter treatment period and a lower rate of treatment failure, compared to non-LED light sources [25,26,27,28]. Phototherapy using optical fibers includes devices such as Bilibed, Bilisoft Cover, Bilipad, or Bilicocoon. Treatment with a conventional phototherapy device with halogen lamps was compared to a light-emitting diode in the BiliCocoon Bag device. The results showed that BiliCocoon Bag technology was a safe and acceptable alternative to providing intensive phototherapy for neonates in the maternity ward that also prevented hospitalization and the separation of mother and child [29]. The possibility of giving home phototherapy improved this relationship and reduced the stress of the parents compared to standard treatment in hospital [30].

### 3.7. Prophylactic or Preventive Phototherapy

Prophylactic phototherapy is defined as phototherapy initiated before bilirubin reaches pre-specified threshold levels. Compared to standard phototherapy, preventive phototherapy reduces the last recorded bilirubin level, and the probability of neurodevelopmental disorders. However, it increases the duration of the phototherapy treatment [31]. A Cochrane systematic review (2012) investigating phototherapy administered prophylactically to premature infants stated that the application of phototherapy shortly after birth can help to avoid the need for exchange transfusion and reduces the risk of central neurological involvement. However, the authors emphasized the very small number of well-designed studies evaluating the long-term effects of this treatment [32]. Interestingly, phototherapy may weaken the protective antioxidant effect of bilirubin on cell membranes, making them more susceptible to injury that could result in cell apoptosis. Boskabadi et al. found that higher bilirubin levels in preterm newborns may act as a protective factor against ROP [33]. Phototherapy itself causes oxidative stress. Thus, there are conflicting interests; while preventing high levels of free serum bilirubin that could cause neurotoxicity is desirable, keeping bilirubin levels at a mildly elevated concentration may be desirable due to their antioxidant function in protecting cell membranes. Extremely low concentrations of bilirubin may even be harmful [34]. In addition, recent evidence has indicated that phototherapy may cause several adverse reactions, including DNA changes and potential cancer development [35]. In general, initiating prophylactic phototherapy before bilirubin levels reach the high-risk threshold and thus prolonging exposure is not recommended.

### 3.8. Possible Complications of Phototherapy

In recent years, data has suggested that phototherapy may induce various adverse reactions. The negative sequelae of providing treatment for jaundice through phototherapy can be divided into mother–newborn interactions, short-, and long-term effects.

#### 3.8.1. Mother–Newborn Interactions

It is well-known that the bonding formed between mothers and their infants after labor is a major factor contributing to their well-being, psychological, and neurological development. Many of the currently available treatments for neonatal jaundice require separating newborns from their mothers. Except in cases of severe jaundice, phototherapy should be intermittently discontinued to allow for breastfeeding, skin-to-skin care, or other types of mother–infant interactions that are important also for reducing parental anxiety [36].

#### 3.8.2. Short-Term Effects

- Thermal regulation and hydration

Changes in circulation have been described during phototherapy treatment in newborns, such as increased blood flow to the skin, which causes a redistribution of the blood [21]. Hyperthermia and dehydration may occur during phototherapy, mainly in premature babies but also in full-term newborns, especially in non-LED photo devices [37]. The use of blue-green LED light may decrease the risk for hyperthermia, but may increase events of hypothermia [38]. On the other hand, the findings of Yassin et al. showed that the use of LED phototherapy to treat premature jaundice increased the average incubator roof temperature by 2.9 °C and the indoor air temperature by 1 °C [39].

Increased blood flow through the skin may cause increased transepidermal water loss: the heating effect of phototherapy causes vasodilation and local heating that increases water loss from the body surface. Hyperperfusion of the skin and a subsequent redistribution of blood occurs [40]. In addition, the excessive breakdown of bilirubin, which is secreted through the intestine, may stimulate the intestinal wall and change the trans membrane potential difference across the epithelium [41], inducing diarrhea with the resultant loss of water, sodium, and potassium [42].

- Electrolyte disturbances

Phototherapy may cause hypocalcemia, perhaps explained by its effects on decreasing melatonin levels and increasing cortisol levels. In addition, PTH suppression was tested and found to be one of the causes of phototherapy-induced hypocalcemia [43]. A comparative study was performed between blue light and conventional white light, and it was found that light-emitting diode phototherapy causes hypocalcemia similar to conventional lamps [44].

- Hematologic effects

Phototherapy can cause hemolysis, possibly through increased oxidative stress in jaundiced neonates, which may decrease antioxidant levels, leading to an imbalance of the body’s oxidative defense system, creating free radicals with the formation of methemoglobin [45]. Another interesting explanation found in studies from earlier years can help understand this phenomenon. The researchers speculated that, since the wavelength of blue light absorption by riboflavin is similar to that of bilirubin, both of them will break down at the same time, which will lead to riboflavin loss in the body. The paucity of riboflavin will decrease the synthesis of active riboflavin adenine dinucleotide, diminish the hydrogen supply of erythrocytes, decrease glutathione reductase, and impair the activity of erythrocyte glutathione reductase, thus exacerbating hemolysis [46]. Light-induced thrombocytopenia has previously been described. Sarkar and colleagues found that there was a positive relationship between a drop in platelet count and increased phototherapy duration. The researchers noted that, despite the prevalence of thrombocytopenia following phototherapy it was mostly mild and transient, and of no clinical significance [47]. However, other researchers found a statistically significant increase in the average platelet count after phototherapy (*p* = 0.001). Their study showed that prolonged phototherapy was associated with an elevated average platelet count in jaundiced neonates exposed to phototherapy for over 2 days [48].

- Sleep disturbances

During phototherapy, eye masks are used to prevent retinal injury from high-intensity light. A previous study found that blindfolding for over 24 h during phototherapy in neonates increased plasma melatonin levels, because continued lack of light stimulates pineal metabolic activity [49]. This, in turn, causes a change in the circadian rhythm of newborns. A recent study investigating the relationship between phototherapy in newborns and sleep disorders found that infants in the phototherapy group had shorter sleep times over 24 h compared to those of the non-phototherapy group. In addition, infants with a longer duration of phototherapy were found to sleep less time per day at one month of age [50].

- Skin

Bronze baby syndrome (BBS) is a rare complication that occurs in newborns who have elevated conjugated bilirubin with cholestasis. The mechanism of BBS is unclear and the threshold of conjugated bilirubin beyond which this syndrome occurs is also unknown, because not all infants with cholestasis develop BBS during phototherapy. BBS is considered to be harmless, and pigmentation slowly resolves if exposure to phototherapy is stopped [51]. However, BBS may be an additional risk for the development of kernicterus [52]. Therefore, newborns with mixed (direct–indirect) hyperbilirubinemia who are under phototherapy treatment should be closely monitored and the causes of underlying cholestasis should be excluded [53]. An erythematous or petechial rash may appear in some phototherapy patients, and it usually disappears when the exposure to light is ceased. Petechiae may be related to light-induced thrombocytopenia [54].

- Cardiovascular effects

Phototherapy may cause hemodynamic changes, such as vasodilation in the skin, which is compensated by vasoconstriction of the renal and mesenteric circulations without significantly affecting the cerebral circulation. Furthermore, hypotension and tachycardia have been described [26,55,56]. Contrary to this, another article found a significant decrease in heart rate variability during phototherapy [57]. The effect of phototherapy in full-term newborns on cardiac output and brain and renal blood flow was also studied. The average blood flow velocity in the left pulmonary artery (LPA) measured by pulsed Doppler ultrasound increased significantly after 12 h of exposure and returned to pre-phototherapy values after its cessation [58]. In addition, the time intervals of opening and closing atrio-ventricular valves were significantly decreased during phototherapy and ventricular ejection times tended to be reduced. However, cardiac output and myocardial performance measures, as well as tissue Doppler velocities, were not affected by phototherapy and were similar to those of controls. The coronary arteries’ blood flow velocities and integrals were significantly diminished during phototherapy in one study [59,60]. However, the researchers concluded that, while some of the findings may indicate a lower flow in the coronary arteries during phototherapy, these changes were not clinically significant in healthy newborns [60]. Additionally, cardiorespiratory activity was influenced by phototherapy during active sleep but not during quiet sleep in term infants with physiologic jaundice. However, the clinical significance of these findings is not known [61].

The functional closure of the patent ductus arteriosus (PDA) typically occurs immediately after birth. However, failed closure may occur in infants receiving phototherapy. A prospective study examining the relationship between phototherapy and PDA reported that phototherapy significantly increased the incidence of PDA among infants with an extremely low birth weight [62]. An attempt to prevent the effect of phototherapy on the ductus arteriosus was recently investigated by a group of researchers who tested the possibility of a chest protector during phototherapy in premature babies younger than 30 weeks of gestation and weighing less than 1000 g. It was found that the hemodynamically significant PDA ratio (hs-PDA) and left atrial to aortic root ratio decreased significantly in the protected group, and the need for PDA treatment was significantly lower. The light treatment times of the two groups were similar and the researchers concluded that the application of shielding reduces the incidence and severity of hs-PDA in very premature infants receiving phototherapy [63].

- Gastrointestinal effects

There is evidence that phototherapy can significantly affect the probiotic flora and thus the metabolites in the gut of newborns, which could explain the side effects of phototherapy in the digestive system. It may also provide a theoretical rationale for giving probiotics to newborns with jaundice as discussed below [64]. The distension of the abdomen and bile stained gastric aspirates are frequent in high-risk neonates undergoing phototherapy. A retrospective study reported that phototherapy is associated with a higher rate of intestinal obstruction in infants with a markedly low birth weight [65]. Another study suggested that increased exposure to phototherapy may increase the risk of developing necrotizing enterocolitis (NEC) in very-low-birth-weight (VLBW) infants. Researchers have shown that the odds for NEC increase with the length and number of phototherapy exposures. They suggested that exposure for more than 120 h and more than four episodes of phototherapy could be significantly associated with NEC in a multivariate analysis [66]. A possible association between phototherapy and NEC was also previously suggested by our group [67].

- Survival

A higher risk of mortality was found with prolonged phototherapy, especially in premature infants weighing less than 750 g [66]. Another study also found that, for infants with birth weight from 501 to 750 g, phototherapy was associated with decreased survival and may counteract any potential benefits of aggressive phototherapy [68]. A longer duration of phototherapy and increased oxidative stress may be important factors associated with higher mortality rates [22,69].

#### 3.8.3. Long-Term Effects

- A long duration of neonatal phototherapy was positively associated with the risk of allergic disorders, especially food allergies [70].
- In addition, blue light treatment during the newborn period may also be related to other morbidities. Authors confirmed a small increase in the risk of childhood convulsions (especially among boys) who received phototherapy in the neonatal period. They noted that the association was not due to hyperbilirubinemia itself or other known confounding variables [71]. A previous study showed similar results and the authors concluded that sex and gestational age may play important roles in this association [72].
- Recently, questions have been raised about the effect of phototherapy on child development. A Japanese observational cohort study was performed that examined the relationship between phototherapy duration for neonatal jaundice and the risk of neurodevelopmental impairment at 3 years of age. A data analysis of 76,897 infants was performed. Four exposure groups were studied: no phototherapy, short phototherapy (1–24 h), long phototherapy (2–48 h), and very long phototherapy (>48 h). After adjusting for relevant risk factors, a dose–response relationship was identified between the duration of phototherapy and the children’s development at age three, and the differences were significant. A longer duration of phototherapy is a predictor of neurodevelopmental delay, therefore, it is prudent to avoid prolonged periods of phototherapy [73].
- Since the 1970s, there have been reports that phototherapy has a mutagenic effect on prokaryotic and eukaryotic cells [74]. A previous study showed the effects of phototherapy on an increase in the incidence of neonatal melanocytic nevi, which is known to be the most important risk factor for the appearance of melanoma in the skin [75]. It has also been suggested that phototherapy in newborns may increase the risk of hemangioma in infants, and this happens as a result of oxidative stress that damages vascular endothelial cells and stimulates the formation of new blood vessels [76]. Accumulating evidence in recent years has indicated that phototherapy may cause a variety of adverse reactions, including DNA damage, cancer risk, and mortality in VLBW [35,77,78]. Phototherapy may be a possible risk factor for cancer. A recently published review found links between phototherapy in newborns and an increased risk of cancer. Specifically, all hematopoietic cancers, all leukemias, and myeloid leukemias showed statistically significant associations. Phototherapy in newborns, according to these results, may be associated with a 1.2 fold increase in the chance of developing any cancer, 1.5 fold risk for any hematopoietic cancer, 1.4 fold risk for leukemia, and 2.9 fold risk for myelocytic leukemia [35]. In addition, a population-based cohort study in a Canadian medical district with a 10-year follow-up in public hospital care indicated that infants who received phototherapy were at an increased risk for cancer several years after exposure, particularly solid tumors. However, the researchers noted that the associations found were weak, and they failed to confirm a direct effect of phototherapy [79]. A recently published population-based retrospective cohort study that included all babies born after ≥32 weeks’ gestation in a single medical center in Israel between 1988 and 2018 compared the incidence of neoplastic diseases between infants exposed to phototherapy and those who were not. The medical center’s database was crosschecked and the diagnosis was verified with the registry of malignant childhood diseases according to the National Cancer Registry of the Israeli Ministry of Health. The study population included 342,172 infants, of which 18,797 (5.5%) were exposed to phototherapy. According to the researchers’ findings, phototherapy was associated with a significantly increased risk of childhood malignancies and benign tumors. Specifically, phototherapy was associated with hematopoietic cancer and leukemia, but not with solid tumors and lymphoma [78]. The increased risk of cancer in children previously exposed to phototherapy may related to the hyperbilirubinemia itself, phototherapy, or a combination of both [79,80].

To conclude, while phototherapy has been the preferred treatment for neonatal jaundice for many years, many complications and harmful effects from this treatment are now known. Thus, the literature in the last decade has included a multitude of studies describing alternative treatments as an addition to phototherapy.

## 4. Strategies for Shortening Phototherapy

In addition to the adverse effects previously described which may be dose-related, phototherapy in newborns is also associated with a considerable socioeconomic burden, including hospital readmissions, prolonged hospitalizations after birth, and infant–mother separation. Thus, measures that could shorten the duration of phototherapy could be beneficial.

### 4.1. Cycled Phototherapy

The method of routinely administering continuous phototherapy to term and preterm infants is debatable. Since accumulating evidence from multiple reports in recent years has shown the damage from continuous phototherapy treatment, there are studies that have tried to find ways to reduce the time of exposure without reducing the effectiveness of the treatment. Research findings point to the disturbing mortality figures for premature infants weighing less than 750 g exposed to continuous phototherapy. Cycling phototherapy may provide an alternative to continuous phototherapy with a reduced exposure that could be beneficial, particularly for extremely low-birth-weight (ELBW, <1000 g) infants [81]. Another article showed that intermittent phototherapy, defined as alternating phototherapy on and off every hour, is as effective as continuous phototherapy, defined as two hours on and half an hour off, in reducing the total serum bilirubin level in mature infants [82].

### 4.2. Positioning during Phototherapy

Turning infants during phototherapy to increase the exposure of more body skin surface area to the phototherapy light is practiced in many nurseries. However, this practice is not evidence-based. In one study, it was shown that infants treated in the supine position only had a more significant decrease in bilirubin levels and required a shorter duration of phototherapy compared to infants who were turned around [83].

### 4.3. Probiotics

The intestinal microbiota (referring to symbiotic microorganisms in the intestine) is considered to be an “organ” of the human body, which plays an important part in protection against pathogens and the nutrition, metabolism, and immune maturation of newborns [84]. Previous studies have suggested that the uterus should be considered to be sterile and that the primary microbiota are introduced into the newborn’s intestine during birth. Recently, a wealth of evidence has casted doubt on this knowledge. These studies have indicated that the genomic DNA of low-abundance bacteria can be isolated from the meconium and other parts of the fetus and placenta. Today, it is known that intestinal bacteria colonize rapidly from the maternal and environmental microflora within minutes of birth. The foundations for healthy gut microbiota lie in early infancy, starting with facultative anaerobes such as Enterobacteria and Enterococci and continuing with anaerobic species, including Bifidobacterium, Bacteroides, and Clostridium [85].

The effect of probiotics on the treatment of neonatal jaundice is a relatively new research field [86]. In clinical practice, apart from the conventional treatments to lower bilirubin values, treatment with probiotic supplements can help by improving immunity, mainly by regulating bacterial colonies. These colonies can form a biological barrier by selectively binding to intestinal epithelial cells through teichoic acid [87]. A meta-analysis that included data from 13 articles based on 1067 newborns provided evidence that combined routine therapy with supplemental probiotic therapy, consisting of *Bifidobacterium*, *S. boulardii*, *C. butyricum*, probiotic oligosaccharides, and *B. subtilis*, was clearly effective in treating neonatal jaundice. The supplemental probiotics not only improved the treatment outcomes by significantly reducing the total bilirubin and jaundice resolution time, but it also reduced the duration of phototherapy and length of hospitalization, without serious adverse reactions [88,89]. Another study evaluated the effects of probiotic supplementation on neonates with indirect jaundice induced by isoimmunization alone, and showed no differences in the rate of bilirubin reduction in the first 24 h of life. However, the effect of the probiotic occurs only after 36 h of life [90]. A systematic review of randomized controlled trials (RCTs) examining probiotic supplementation for the prevention or treatment of neonatal jaundice (not limited by gestation or birth weight) using the Cochrane methodology showed limited, low-quality evidence that probiotic supplementation may decrease the duration of phototherapy. The authors concluded that the routine use of probiotics cannot be recommended for the prevention or treatment of neonatal jaundice, and large, well-designed trials are essential to confirming these findings. They did note that there were no side effects associated with probiotics [91]. According to different results and a wide palette of different preparations, it is not currently possible to recommend the routine use of probiotics, prebiotics, or symbiotics [92].

### 4.4. Pomegranate Juice

One study examined the consumption of pomegranate juice in mitigating neonatal jaundice. The researchers conducted an RCT study, testing the effect of consuming concentrated pomegranate juice (CPJ) among breastfeeding mothers whose children received phototherapy and breast milk, compared to an identical group of mothers who did not receive pomegranate juice. The results showed reduced bilirubin levels within 48 h for hospitalized breastfed newborns whose mothers consumed CPJ, leading to shorter hospital stays and faster discharge [93]. These effect could be related to the vitamin C and polyphenolic compounds in pomegranate juice that appeared in the breast milk of the mothers after consuming the pomegranate juice. Since the antioxidant ability of neonates is limited, neonates are more susceptible to oxidative damage that can cause hemoglobin breakdown, leading to hyperbilirubinemia. The antioxidant activity transferred from the CPJ to the infant via the mother’s milk can strengthen the infant’s immune system and reduce oxidative reactions [93].

### 4.5. Fibrate

Many previous RCTs have evaluated fenofibrate as an adjunctive treatment to phototherapy in neonates with elevated levels of indirect bilirubin [94,95,96,97]. Fibrate is a class of phenoxy-isobutyric acid derivative, which consists of peroxisome proliferator-activated alpha receptor agonists, used as a lipid-lowering drug that can also enhance bilirubin conjugation and excretion through the induction of UGT1A1. A group of researchers reported that a single dose of fenofibrate at a dose of 10 mg/kg during phototherapy in newborns is associated with a significant reduction in serum bilirubin levels [94]. Another study failed to demonstrate a statistically significant difference in the rate of bilirubin reduction despite the shorter duration of phototherapy [98]. In a systematic review and meta-analysis based on results from five studies, significant effects of fenofibrate as a complementary treatment to phototherapy on reducing serum bilirubin levels were found. However, the meta-analysis did not show a separated effect of fenofibrate, independent of phototherapy, on reducing the bilirubin levels. Thus, the authors recommended further studies [99]. In a recent study, authors reported that combining conventional phototherapy with a single oral dose of 10 mg/kg fenofibrate may shorten the time it takes to lower the bilirubin levels. This supports an effect of fenofibrate as an adjuvant therapy to phototherapy in neonates that may shorten length of phototherapy and accelerate discharge from the hospital [100].

Another drug in this group that was tested was clofibrate. It processes the transfer of albumin-bound bilirubin to hepatocytes and stimulates hepatic uptake and the conjugation of bilirubin. Clofibrate is a fibric acid derivative used in the treatment of hyperlipoproteinemia type III and severe hypertriglyceridemia. The researchers performed a double-blind clinical study on 100 term newborns randomly assigned to one of two groups, in which they evaluated the effect of clofibrate and phototherapy on prolonged breast-milk-derived jaundice in term healthy newborns. The intervention group received clofibrate at 50 mg/kg dissolved in 2 mL of distilled water that was given as a single dose in addition to phototherapy, and the control group received a placebo of the same amount of distilled water with phototherapy. The intervention and control infants were matched. A statistical analysis of the results showed that the bilirubin reduction rate was significantly faster in the intervention group compared to the control group (*p*  <  0.05). The average length of hospital stay and phototherapy in the intervention group was also significantly shorter (*p*  =  0.005). The researchers concluded that clofibrate effectively reduced bilirubin levels and shortened the duration of phototherapy and length of hospital stay in infants with presumed breast milk jaundice [95].

### 4.6. Metalloporphyrins

Since the early 1980s, scientists have worked extensively with synthetic metalloporphyrins consisting of protoporphyrin IX ring-structured macrocycles with other metals. Tin (Sn), zinc (Zn), manganese (Mn), nickel (Ni), magnesium (Mg), copper (Cu), cadmium (Cd), or cobalt (Co) serve as a central atom exchanger for iron (Fe) in the natural heme. This way, they can act as a potent competitive inhibitor of heme oxidation through heme oxygenase. When these metalloporphyrins are present, heme cannot be degraded to bile pigments, i.e., biliverdin and, ultimately, bilirubin [101,102,103]. The scientists formulated certain criteria to identify a convenient metalloporphyrin for the treatment of hyperbilirubinemia in newborns. By definition, the ideal inhibitor should contain a metal that occurs naturally in the body or is not harmful in trace amounts, does not break down in the tissue into toxic substances, adequately inhibits oxygen in relatively low doses, and does not participate in photo-destructive reactions [104]. To this day, there is limited evidence regarding the efficacy and safety in neonates, and synthetic metalloporphyrins are not approved for routine clinical use in many countries, including the USA and the UK. However, tin mesoporphyrin (Sn-mesoporphyrin: Sn-MP) is now under investigation in a phase II clinical trial to compare its prophylactic and therapeutic efficacy in unconjugated hyperbilirubinemia [105,106]. Until now, mixed and insufficient evidence has been received regarding short- and long-term safety, and it is not possible to decide on the clinical use of porphyrins for the treatment of neonatal hyperbilirubinemia. A recent multicenter, placebo-controlled phase 2b study evaluated the efficacy and safety of tinned mesoporphyrin (SnMP) in neonates over 35 weeks’ gestation with hyperbilirubinemia due to hemolysis under phototherapy. The newborns were randomly divided into three equal groups (placebo, SnMP 3.0 mg/kg, or SnMP 4.5 mg/kg) given IM once within 30 min from the start of phototherapy. The researchers noted that, by 48 h, significant decreases in bilirubin values were observed in the SnMP intervention groups (*p*  <  0.0001) compared to the placebo control group, where increases were observed. In the intervention groups, bilirubin significantly decreased by −13% (*p*  =  0.013) in the 3.0 mg/kg and by −10.5% (*p*  =  0.041) in the 4.5 mg/kg group by 48 h [107]. This study provides another step towards the wider use of this group of medications as a possible complementary treatment for neonatal jaundice with hemolysis.

### 4.7. Zinc Sulfate

There are reports on the effectiveness of the combined treatment with phototherapy and zinc sulfate. Zinc sulfate has an antioxidant effect and is also an enzyme co-factor. These properties could be valuable in heme catabolism and bilirubin production [108,109]. Its combination with phototherapy may be beneficial and safe for reducing the level of bilirubin and the duration of phototherapy in term and premature newborns with indirect jaundice [110,111,112,113]. A group of researchers tested and found that phototherapy has no effect on the level of zinc in the serum. However, zinc supplements are encouraged in cases of neonatal jaundice, because they are usually zinc deficient [111]. On the other hand, another group of researchers claimed that it must be taken into account that there could be a risk of raising the level of zinc in the serum during phototherapy. Therefore, giving zinc supplements, together with intensive phototherapy, may cause zinc toxicity, and is thus not appropriate for newborns with high levels of indirect bilirubin in the serum under intensive phototherapy treatment [111]. There is no consensus regarding a recommendation for zinc supplementation in these newborns.

### 4.8. Kangaroo Mother Care

The Kangaroo Mother Care (KMC) method was first described in a hospital in Colombia in 1970s. KMC involves the skin-to-skin care of the newborn in the kangaroo position at birth or shortly after [114]. Studies have shown that KMC facilitated a reduction in neonatal morbidity and mortality among low-birth-weight premature infants [115,116]. It was reported that jaundiced neonates undergoing KMC needed a shorter duration of phototherapy. Also, phototherapy together with intermittent KMC helped to reduce the overall duration of phototherapy [117,118,119].

### 4.9. Massage

Recently, massage therapy has been introduced as a new and effective method in the treatment of newborns with jaundice [120]. Shahbazi et al. showed that the average bilirubin levels decreased as the level of massage intervention (i.e., massage duration and frequency per minute) increased. They suggested a reversed linear relationship between massage therapy and neonatal jaundice [121]. The phenomenon can be explained by the fact that the massage performed on the newborn’s body stimulated the movement of blood and lymph, and this promoted the dynamic migration of bilirubin and reduced its reabsorption through the enterohepatic circulation [122]. Other researchers showed that a massage effectively reduced serum bilirubin levels, increased stool frequency, the proportion of milk consumption, and weight gain, and shortened the duration of phototherapy [123].

### 4.10. Traditional Chinese Medicine

#### 4.10.1. Tuina

According to traditional Chinese medicine, the practice of Tuina, also called Tui Na, implying “push (and) grip”, that has been utilized in China for many centuries could be beneficial. It is a non-medicinal, manual treatment, which is mainly applied to the meridians or acupuncture points, which are pathways for the qi (energy moving in the meridians and the muscles) and blood of the human body, by pushing, holding, pressing, and rubbing [124]. Tuina in combination with blue light therapy for the treatment of neonatal jaundice can enhance the effect of clinical treatment and reduce the side effects caused by continuous blue light therapy [125].

#### 4.10.2. Medical Bath

In the bath method of traditional Chinese medicine, substances have body-purifying and detoxifying effects. A newborn is placed in a bucket with water at a temperature of 37–38 degrees Celsius with the addition of various plant waters. Each plant has its own effect. A traditional Chinese medical bath is simple to operate, and there is no need to use special medical devices or equipment. The treatment is based on using common materials and is low cost. Soaking children in the medicine bath bucket creates a large area of contact with the medicine liquid. The hot steam can open the pores in the children’s skin and promote the penetration of the medicine liquid. In addition, the traditional Chinese medicine bath program can promote blood circulation in the body, improve metabolism, and accelerate the absorption of drugs. The combination of phototherapy and bath in traditional Chinese medicine accelerates the secretion of bilirubin in newborns. Researchers decided to test the effect of combining phototherapy with a heated Chinese bath. Before treatment, the jaundice index of both groups was equivalent to the serum bilirubin level. After the treatment, the study group showed a greater improvement than the control group. Also, the time to the disappearance of jaundice symptoms and the length of hospital stay in the study group were shorter compared to the control group. The results showed that the use of traditional Chinese medicine baths as complementary treatment for neonatal jaundice can achieve a good therapeutic effect, promote the rapid disappearance and remission of the symptoms of the disease, and accelerate the secretion of bilirubin, and is thus considered to be a method with a high therapeutic value and good safety [126].

#### 4.10.3. Oral Herbal Treatment

Herbal treatment of neonatal jaundice has been practiced in China for a long time. Yinzhihuang, consisting of extracts of Artemisia capillaris Thunb., Gardenia jasminoides Ellis, Lonicera japonica Thunb., and Scutellaria baicalensis Georgi, is a well-known traditional Chinese potent medicine for patients with liver disease used in China. There are many misconceptions and unfounded beliefs about the relationship between herbal medicine and the treatment of neonatal jaundice. Yet, traditional Chinese medicine has been used for almost 3000 years and its beneficial effects are still being studied and integrated into conventional treatments in modern times [127]. Complementary herbal treatments for the treatment of hyperbilirubinemia in newborns have recently come into use. Traditional herbal medicines may play a significant role in reducing the serum bilirubin level by increasing the needs and stopping the enterohepatic cycle. In the professional literature from China, there are few works examining the administration of Yinzhihuang oral liquid in combination with phototherapy for the treatment of neonatal jaundice. The methodological quality of the trials was assessed according to the Cochrane Collaboration’s risk of bias tool. A total of 17 trials (including 2561 newborns) were described. Side effects were reported in eight trials, but none of them were serious. Based on trials with a low methodological quality, Yinzhihuang oral liquid in combination with phototherapy appears to be safe and superior to phototherapy alone for reducing the serum bilirubin in neonatal jaundice [128]. A later systematic review supported previous reports that Yinzhihuang given to neonates along with phototherapy is more effective than phototherapy alone in reducing indirect bilirubin values [129].

### 4.11. Folk Traditions, Beliefs, and Superstitions

There are multiple folk beliefs regarding “treatments” for jaundice in newborns. Some of these practices are harmless and involve dressing a newborn in yellow or green clothes, placing yellow or green objects like apples, or even putting real gold inside the baby’s cradle. Others can be harmful and involve the use of animals, such as putting the newborn’s feet in a tub with a live goldfish or placing a live pigeon on the newborn’s belly. As even a broken watch is accurate twice a day, similarly, folk treatments sometimes coincide with natural bilirubin reduction, leading families to believe in their miraculous efficacy. However, folk beliefs can occasionally be based on active mechanisms of substances that have been traditionally used. In this part of the review, we describe popular and traditional beliefs about treatments that have passed down from generation to generation for hundreds of years and have recently been tested in studies and found to be effective.

#### Purgative Manna

Purgative Manna, an ancient treatment for neonatal jaundice in Iran, was known long before phototherapy became common practice. Additional names in use are Cotoneaster and Shirksht. Manna Purgative, a sweet, white, and slightly yellow substance produced by an insect of the genus Cotoneaster of the Rosacea family, contains mannitol, fructose, glucose, and sucrose as its main ingredients. It is considered to be a mild laxative that can remove toxins from the bile ducts, liver, stomach, and gallbladder [130] Iranian researchers examined its use as a complementary medicine in the treatment of neonatal jaundice alongside phototherapy, and reported that the purgative manna’s laxative effect shortened the duration of phototherapy, probably by reducing the reabsorption of bilirubin through the enterohepatic circulation [131,132]. There is also a description from the old European literature of a Swedish doctor, Nils Rosen von Rosenstein (1706–1773), who recommended giving a laxative in the form of ground manna sugar powder to treat newborn jaundice. Rosen von Rosenstein’s views and approach regarding neonatal jaundice were identical to those found in other medical texts from the 18th and 19th centuries [133].

### 4.12. Enhancement of Defecation

Oral agars can decrease enterohepatic circulation by enhancing stooling and meconium evacuation. However, they have not proven useful for preventing significant neonatal jaundice [134]. A recent study on the use of oral agar given in combination with phototherapy showed that it was more effective in treating neonatal hyperbilirubinemia compared to phototherapy alone, and reduced the duration of phototherapy [135,136]. This raises the question of whether other means of enhancing stooling, such as glycerin suppositories, could help in the evacuation of meconium, thus decreasing the enterohepatic recirculation of bilirubin. The researchers examined the repeated administration of glycerin suppositories immediately after birth until the evacuation of the first stool. Although glycerin caused a faster meconium evacuation, it had no consistent effect on preventing jaundice or decreasing bilirubin levels at 48 h of life compared to controls. They did not check whether it could be beneficial as a complementary treatment with phototherapy [137]. A meta-analysis that examined the use of glycerin suppositories and enemas in premature infants concluded that it was indeed associated with the earlier evacuation of meconium, but the clinical significance of this finding was unclear [138]. Nevertheless, another meta-analysis that examined the effects of glycerin suppositories on time to full enteral feeds in preterm infants and included five RCTs with 266 premature infants found that glycerin suppositories may actually increase the days of phototherapy compared to control group [139].

## 5. New Technologies

New novel innovative treatments should be developed and tested in order to decrease NHB while preventing harm to newborns. Recently, newborn genomic sequencing (nGS) has brought about novel opportunities that could further expand newborn screening (NBS), which is an important public primary care preventive health project. A new study demonstrated the possibility of the clinical evaluation of neonatal genomic screening for infants in neonatal intensive care units (using neonatal hyperbilirubinemia as an example). A total of 96 newborns with jaundice were examined. The researchers collected dried blood spots 72 h after birth. At the same time, tandem mass spectrometry (TMS) testing and Angel Care genomic screening (based on next-generation targeted sequencing) were performed. Many associations were found between hyperbilirubinemia and various other disorders, such as maple syrup, autosomal recessive deafness, and thyroid hormonogenesis disorders. In addition, 44 infants (45.8%) were identified with at least one variant conferring a carrier status to a childhood recessive disorder. This study, using neonatal hyperbilirubinemia as an example, showed that sequencing the genome can find many genetic variations that might provide future directions for adjunctive treatments [140].

Studies revealing important genetic associations that could be related to significant NHB, such as uridine diphosphate glucuronosyltransferase isoenzyme (UGT1A1) gene variants and their role in breastmilk jaundice [141], might prove useful in developing new therapeutic complementary interventions that could possibly decrease exposure to phototherapy without hampering infants’ safety or the desired effect of decreasing bilirubin levels.

## 6. Discussion

Our desire to prevent chronic bilirubin encephalopathy through active surveillance and early treatment with phototherapy has led to the fact that hyperbilirubinemia is the most common diagnosis after birth and is also the most common reason for re-hospitalization during the first week of life. Our review of the literature showed that providing phototherapy for neonatal jaundice, although considered to be relatively safe, is not free from side effects and possible short- and long-term adverse outcomes. This issue is complicated, because the effects of phototherapy are affected not only by its duration, but also by the intensity and type of light used on the exposed skin area of the newborn baby. We did not find a study that provided definite answers to the questions of what the preferable approach is to phototherapy intensity or if the high phototherapy intensities, as recommended today (of at least 30 µW/cm^2^ per nm) [142], are less or more harmful compared to the lower phototherapy intensities that were used in the past (5–10 µW/cm^2^ per nm). Allegedly, powerful phototherapy lowers the bilirubin levels quickly, thus shortening the duration of the treatment and its harmful effects. However, it is possible that lower intensities (although for longer durations) might be associated with less adverse outcomes.

Although phototherapy is the accepted standard of care for hyperbilirubinemia, the thresholds for initiating phototherapy have continued to vary over the years. The American Academy of Pediatrics (AAP) Guidelines from 2004 [143] definitely used lower threshold levels for treatment compared to the new published guidelines from 2022 [6]. In 2016, the Northern California Neonatal Consortium (NCNC) published their first phototherapy guidelines, which were updated in 2017 [144], and the latest 2022 AAP guidelines [6] clearly follow them, suggesting to start phototherapy at slightly higher bilirubin thresholds. Recent evidence for the safety in choosing these higher bilirubin thresholds for initiating phototherapy comes from an observational study [145] that assessed the transition from using the 2004 AAP guidelines [143] to the 2019 NCNC guidelines [144] in healthy infants born after 35 or more weeks of gestation. The authors found that higher thresholds for phototherapy treatment were associated with a decrease in phototherapy rate from 6.4% to 4%. However, there were no significant changes in incidences of bilirubin at >25 mg/dL or bilirubin within 2 mg/dL of the exchange transfusion thresholds [145]. These indicators for safety were based on many studies of cases of kernicterus. These studies showed normal developmental outcomes in infants at >35 weeks gestation and without hemolytic disease with bilirubin levels above 25 but below 30 or within 2 mg/dL of the infusion threshold [6,71,144]. A recent study using the new guidelines also showed that there were no changes in the rates of exchange transfusion or readmission for phototherapy between the period before and after the change [145]. The authors concluded that raising phototherapy thresholds can decrease the need for phototherapy without increasing critical hyperbilirubinemia or readmission rate [145]. Thus, adherence to treatment guidelines can also be considered as a measure for preventing excess exposure to phototherapy.

It is important to bear in mind that, despite all its potential risks and side effects, phototherapy has saved many lives and prevented many cases of severe brain injuries. Therefore, this effective, accessible, readily available, and relatively low-cost and safe treatment for neonatal jaundice will continue to be a necessary, irreplaceable, and important cornerstone in the management of NHB. However, in light of the accumulating evidence of its possible side effects and short- and long-term outcomes, it is important to consider adjunct treatments in order to limit exposure to phototherapy to what is mandatory. As discussed in our review, other measures, including those from complementary or alternative medicine, provide an effective means for reducing the duration of phototherapy in newborns with hyperbilirubinemia. Currently, their use should be considered as complementary to phototherapy.

## 7. Limitations

This review has several limitations. First, although we tried to focus on publications from the last decade, the distribution of the publication years of the cited relevant articles was wide in some of the subjects discussed. While some of the studies were based on randomized controlled trials, others were observational. Also, we could not address or assess the quality of many of the studies. Lastly, our search was limited to publications in English, thus, we probably missed some relevant studies, especially in relation to traditional and Chinese medicine.

## 8. Conclusions

Phototherapy has been the preferred treatment for neonatal jaundice for many years. From the time it was first introduced as a treatment for neonatal jaundice, phototherapy has proven to be a very effective and accessible therapy that has prevented many cases of kernicterus and saved millions of children from severe brain damage and even death, thus contributing more than most treatments in neonatology to saving lives and maintaining a good quality of life. Although considered as safe, it is not free from possible immediate and future mild and severe side effects, but most complications of phototherapy are relatively mild or rare compared to other procedures. However, since the possible complications from prolonged exposure to phototherapy lamps are known today, it is recommended to invest efforts into finding new ways to treat hyperbilirubinemia. In the future, it might be possible that phototherapy will be replaced by a new treatment that has not yet been found. Until new technologies come into use and prove themselves to be at least as effective and less harmful, phototherapy will continue to be used as the main treatment for NHB, while strictly following the indications for treatment, avoiding unnecessary overexposure and being aware of the possible consequences. It seems that, currently, the most likely progress will be to introduce new forms of treatment combinations in order to find ways to reduce the duration of exposure to phototherapy without compromising the its effectiveness. There is a need for scientific studies to test combinations of conventional and alternative treatments in order to find the most effective combination without harmful effects that could reduce exposure to phototherapy, and thus the side effects described in this review.

## Data Availability

Not applicable.
